# Outcomes of Discectomy by Using Full-Endoscopic Visualization Technique via the Transcorporeal and Transdiscal Approaches in the Treatment of Cervical Intervertebral Disc Herniation: A Comparative Study

**DOI:** 10.1155/2020/5613459

**Published:** 2020-05-30

**Authors:** Youliang Ren, Junsong Yang, Chien-Min Chen, Kaixuan Liu, Xiang-Fu Wang, Jian-Min Wei, Lei Shi, Wen Liu, Haitao Jiang, Hang Zhou, Shen Li, Zhou Xu, Wanqian Zhang, Zhongliang Deng, Lei Chu

**Affiliations:** ^1^Department of Orthopaedics, The Second Affiliated Hospital, Chongqing Medical University, No. 76 Linjiang Road, Yuzhong District, Chongqing, China; ^2^Department of Spinal Surgery, Honghui Hospital, Medical College of Xi'an Jiaotong University, No. 76 Nanguo Road, Beilin District, Xi'an, Shanxi, China; ^3^Division of Neurosurgery, Department of Surgery, Changhua Christian Hospital, Changhua, Taiwan; ^4^School of Medicine, Kaohsiung Medical University, Kaohsiung, Taiwan; ^5^College of Nursing and Health Sciences, Dayeh University, Taiwan; ^6^Atlantic Spine Center, 475 Prospect Ave, West Orange, NJ 07052, USA; ^7^Department of Spinal Minimally Invasive Surgery, Gansu Provincial Hospital of Traditional Chinese Medicine, No. 418 Guazhou Road, Qilihe District, Lanzhou, Gansu, China; ^8^Department of Spine Surgery, Baoji City Hospital of Traditional Chinese Medicine, Baoji, Shaanxi, China; ^9^Department of Orthopaedics, The First Affiliated Hospital, Gansu University of Chinese Medicine, No. 418 Guazhou Road, Qilihe District, Lanzhou, Gansu, China

## Abstract

**Objective:**

To compare the difference in clinical and radiographic outcomes between anterior transcorporeal and transdiscal percutaneous endoscopic cervical discectomy (ATc-PECD/ATd-PECD) approaches for treating patients with cervical intervertebral disc herniation (CIVDH).

**Method:**

We selected 77 patients with single-segment CIVDH and received ATc-PECD or ATd-PECD in the Second Affiliated Hospital of Chongqing Medical University between March 1, 2010, and July 1, 2015. 35 patients suffered from ATc-PECD, and there were 42 patients in the ATd-PECD group. Obtaining the data of 1, 3, 6, 12, and 24 months postoperatively, the VAS for neck and arm pain and the modified MacNab criteria were used to evaluate the clinical outcomes, comparing radiographic outcomes and complications of these two groups.

**Results:**

We found that the mean operative time was significantly longer in the ATc-PECD group (*P* < 0.05). At the 2-year follow-up, the mean VAS score for neck and arm pain was significantly decreased in both two groups. There was no significant difference in the VAS score for arm pain and neck pain between the two groups at the 2-year follow-up (*P*=0.783 and *P*=0.785, respectively). For the ATc-PECD group, the difference in the height of IVS or vertebral body was significant between the preoperative and postoperative groups (*P* < 0.05, respectively). For the ATd-PECD group, there was only a significant decrease in the height of the IVS (*P* < 0.05); the decrease in the surgical vertebral body was not significant between the preoperative and postoperative groups (*P* > 0.05).

**Conclusion:**

In the 2-year follow-up, there is no significant difference in the clinical outcomes between the 2 approaches. While the longer time was consumed in the ATc-PECD group, the lower rate of disc collapse and recurrence is notable. Additionally, when the center diameter of tunnel was limited to 6 mm, the bony defect can be healed without the occurrence of the collapse of the superior endplate, and ATc-PECD may be preferable in the endoscopic treatment of CIVDH.

## 1. Introduction

Currently, anterior cervical discectomy and fusion (ACDF) is the gold standard for treating cervical intervertebral disc herniation (CIVDH) due to its relatively good safety and efficacy [1–3]. However, with the continuous popularization of this technique, fusion surgery complications, especially the adjacent segment degeneration, have gradually received attention [4–9]. To further prevent these disastrous post-ACDF complications, the exploration of new surgical techniques and the improvement of surgical approaches are continuous.

In recent years, the application of spinal endoscopy has become more prevalent. Spinal endoscopy creates a bridge between conservative treatment and traditional open surgery and has been successfully applied in the treatment of cervical degenerative diseases [10–14]. At present, percutaneous endoscopic cervical discectomy (PECD) is mainly divided into the anterior transdiscal approach and the posterior interlaminar approach [12, 13]. Although the anterior transdiscal approach can provide direct decompression for central or paramedial CIVDH, the iatrogenic disc damage may result in decreased intervertebral space (IVS). This phenomenon has been confirmed by some scholars [12, 14]. In our previous comparative study, we found that compared to the posterior interlaminar approach, the postoperative reduction of IVS was more significantly effective with the anterior intervertebral approach [15]. In order to preserve the intervertebral nucleus pulposus as well as possible, we introduced the anterior transcorporeal approach into the cervical endoscopic spine surgery and observed the satisfactory clinical outcome [16]. Theoretically, the effect on the IVS was controlled after the anterior transcorporeal PECD (ATc-PECD). However, the complication of postoperative vertebral body collapse was also noted. If we reduce the size of bony tunnel, is there any difference in the clinical outcome when compared to the anterior transdiscal PECD (ATd-PECD)? Facing all above problems, we first compared the difference in clinical and radiographic outcomes between ATd-PECD and ATc-PECD in this study.

## 2. Materials and Methods

This retrospective study was approved by the Ethics Committee of the Second Affiliated Hospital of Chongqing Medical University. A total of 91 patients with single-segment CIVDH received ATc-PECD or ATd-PECD in the Second Affiliated Hospital of Chongqing Medical University between March 1, 2010, and July 1, 2015. All above operations were performed by the same surgeon (DZL), who own the experience of over 1000 percutaneous endoscopic lumbar surgeries and 100 posterior endoscopic cervical surgeries. The inclusion criteria included the following: (1) failed conservative therapy for at least 6 weeks or symptom deterioration to the extent of being unbearable, (2) a ventral IVS height of ≥4 mm, (3) neurological symptoms consistent with preoperative MRI, (4) single-level central or paramedian disc herniation, and (5) complete follow-up data of the 2-year follow-up. The exclusion criteria included the following: (1) downward migration of the herniation at the C6-C7 level, (2) disc herniation accompanied by foraminal stenosis or lateral disc herniation, (3) cases of severe obesity accompanied by a short neck with a visceral sheath that was difficult to push to the opposite side, (4) disc herniation with severe calcification, (5) definite instability of the cervical spine (horizontal displacement >3.5 mm or angular displacement >11°), and (6) without complete follow-up data of the 2-year follow-up. According to the inclusion and exclusion criteria, 77 patients were enrolled in the study. The first 35 cases of them belonged to the ATc-PECD group; the following 42 patients treated by ATd-PECD were classified as the ATd-PECD group.

### 2.1. Operational Technique

**Figure 1:**
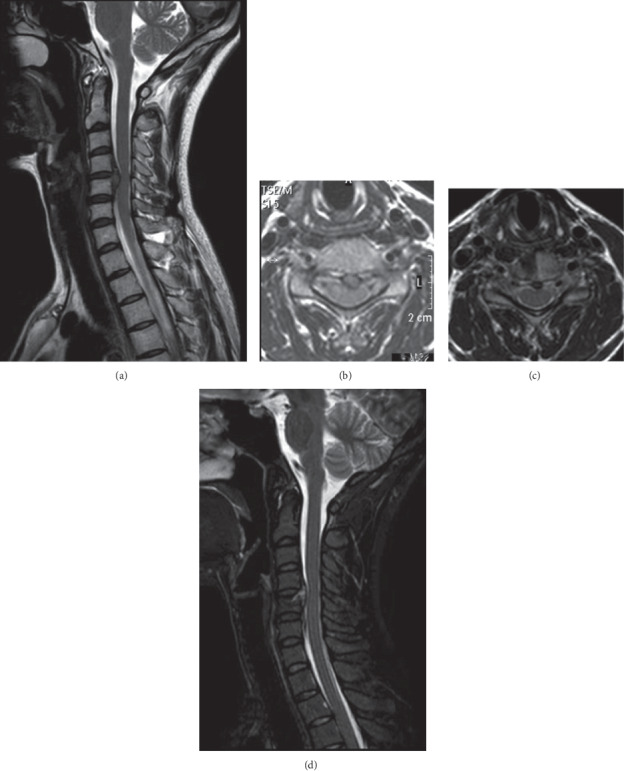
(a) T2-weighted sagittal magnetic resonance image (MRI) shows a central herniated disc behind the C5 vertebral body. (b) The axial MRI scan shows the herniated disc compresses the spinal cord and migrated downward. (c) Axial MRI after ATc-PECD (note the drilling tunnel in the C4 vertebral body). (d) Sagittal MRI after ATc-PECD shows a removal of the protruded nucleus pulposus.

**Figure 2:**
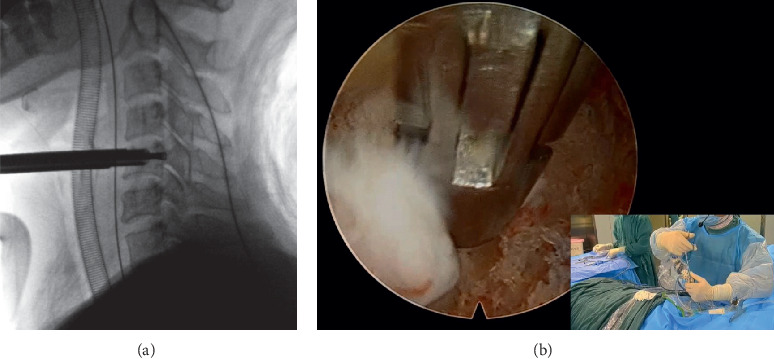
(a) Intraoperative fluoroscopy shows that the working cannula has been satisfactorily assembled. (b) The herniated disc is removed by the endoscopic rongeur under endoscopy.

**Figure 3:**
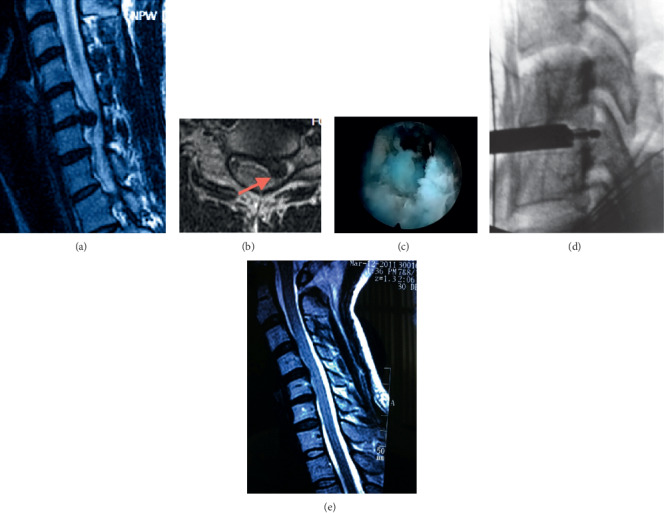
(a) T2-weighted sagittal magnetic resonance image (MRI) shows a central herniated disc at the level of C5-C6. (b) The axial MRI scan shows the herniated disc compresses the left spinal cord and nerve root. (c) The herniated disc was visible under endoscopy. (d) Fluoroscopy shows that the working cannula has been satisfactorily placed at the C5-C6 disc. (e) Sagittal MRI after ATd-PECD shows a removal of the protruded nucleus pulposus.

The operations were performed under general anesthesia with electroneurophysiological monitoring. The right side approach had been utilized for the patients with central disc herniation. The contralateral approach had been applied to deal with the paramedian cervical herniations. The detail of surgical process including location and puncture was similar with our previous studies [15, 16]. The 2-finger technique was used to facilitate the disc puncture [15]. During the puncture process, the lateral carotid artery and the medial tracheoesophagus were pushed toward the opposite side, and the tissue space between them was enlarged with the index and middle fingers of the left hand. This creates a small safe window between the tips of these 2 fingers for the insertion of the spinal needle where the anterior edge of the target disc or vertebral body is perceived. The puncture needle was inserted and passed successively through the following structures: the cervical fascia between the carotid artery (laterally) and the tracheoesophagus (medially), the anterior longitudinal ligament, into the anterior annulus fibrosus or vertebral body inside the window between the bilateral longus colli muscles. After the depth of the tip of the guide wire reached, it was replaced by the puncture needle. The working cannula was placed into the disc space via the transdiscal approach or within the vertebral body via the transcorporeal approach toward the targeted lesion (see Figures 1–3). Among the patients of the ATd-PECD group, under the direct vision of an endoscope, the dorsal annulus fibrosis and posterior longitudinal ligament (PLL) were opened together. Thus, the extruded disc material was exposed and excised using a rongeur. For the patients from the ATc-PECD group, a diamond high-speed burr (SPINENDOS Drill system; SPINENDOS GmbH, Munich, Germany) was utilized to enlarge the bony tunnel within the vertebra. The PLL between the instruments and neural structures was incised using basket forceps and a Kerrison rongeur, after which the extruded disc material was precisely identified. The blunt hook palpation along the surface of the posterior spinal cord was used to determine whether the neural decompression was thorough. Once meticulous hemostasis was achieved, only for the patients from the ATc-PECD group, a drainage tube was retained in the bony tunnel for 24 hours to avoid the possibility of hematoma. All patients were advised to wear a neck collar for 2 weeks to ensure that the damaged annulus fibrosis could be favorably healed decreasing the recurrence of disc herniation.

### 2.2. Follow-Up

During follow-up at 1, 3, 6, 12, and 24 months postoperatively, the VAS for neck and arm pain and the modified MacNab criteria were used to evaluate the clinical outcomes [17]. Additionally, the cervical lateral radiography at the neutral position was utilized to measure the height of the IVS and the vertebral body was recorded, respectively, for each patient. The dynamic radiography was applied to evaluate the stability of the cervical spine. Cervical MRI was performed on a random sample of patients with excellent or good outcomes and in all patients with fair or poor recovery.

### 2.3. Statistical Analysis

The independent samples *t*-test and Chi-square test were performed to compare whether the differences in the indicators between the two groups were statistically significant. A probability level of less than 0.05 was considered to be the threshold of significance.

## 3. Results

### 3.1. Clinical Outcomes

The demographic data and treatment level are shown in Table 1. A significantly higher mean operative time was observed at the ATc-PECD group than that of the ATd-PECD group (87.5 ± 16.8 mins VS 63.5 ± 10.5 mins, *P* < 0.05). At the 2-year follow-up, the mean VAS score for neck and arm pain was significantly decreased in both two groups. For the ATd-PECD group, the mean VAS score for arm pain was improved from 6.1 ± 0.5 to 1.1 ± 0.2 (*P* < 0.001); the mean VAS score for neck pain was improved from 3.7 ± 0.1 to 2.6 ± 0.1 (*P* < 0.001). For the ATc-PECD group, the mean VAS score for arm pain was improved from 6.3 ± 0.6 to 1.1 ± 0.3 (*P* < 0.001); the mean VAS score for neck pain was improved from 3.7 ± 0.1 to 2.6 ± 0.1 (*P* < 0.001) (Table 2). There was no significant difference in the VAS score for arm pain and neck pain between the two groups at the 2-year follow-up (*P*=0.783 and *P*=0.785, respectively) so as to the excellent or good results (32/35, 91.4% vs. 39/42, 92.9%) at the 2-year follow-up.

### 3.2. Radiographic Outcomes

For the ATc-PECD group, mean height of IVS was 6.8 ± 0.4 mm preoperatively, which decreased to 6.3 ± 0.5 mm at the 2-year follow-up (7.4% decrease). The preoperative height of the surgical vertebral body also decreased from 15.8 ± 0.5 mm to 15.1 ± 0.4 mm at 2 years postoperatively (4.4% decrease). The difference in the height of IVS or vertebral body was statistically significant between the preoperative and postoperative (*P* < 0.05, respectively) groups (Table 2). For the ATd-PECD group, mean height of IVS was 6.0 ± 1.5 mm preoperatively, which decreased to 5.1 ± 1.1 mm at the 2-year follow-up (15% decrease). The preoperative height of the surgical vertebral body also decreased from 15.1 ± 0.4 mm to 15.0 ± 0.2 mm at 2 years postoperatively (0.7% decrease). While there was a significant decrease in the height of the IVS (*P* < 0.05), the surgical vertebral body between the preoperative and postoperative groups was not significant (*P* > 0.05) (Table 2), and no instability formation was observed at the final follow-up. The satisfactory neural decompression was confirmed at the postoperative MRI. For the ATc-PECD group, the bony heal of the transcorporeal drilling tunnel was observed on CT scans (fully healed in 33 patients and partially healed in 2 patients).

### 3.3. Complications

No severe complications such as major vessel injury, spinal cord, esophagus injury, or infection were observed in two groups. Two patients belonged to the ATd-PECD group experienced the reherniation and lost to further follow-up. Except the recurrence, a slightly higher rate of complication was noted at the ATc-PECD group (3/35, 8.6%) than that of the ATd-PECD group (2/42, 4.8%). Among the patients from the ATc-PECD group, one patient experienced mediastinal effusion and the other two patients developed postoperative superior-middle endplate collapse without radiological instability. In the ATc-PECD group, one patient complained of a temporary postoperative headache. Another patient developed a postoperative hematoma, which was revised by the ACDF.

## 4. Discussion

In recent years, minimally invasive spinal surgery has achieved great developments. To avoid fusion surgery complications such as ACDF, spinal nonfusion technologies represented by PECD, and artificial disk replacement (ADR) have become hot areas of current research. However, spontaneous fusion and heterotopic ossification after ADR remain as issues of ADR that need to be solved. In the previous studies, PECD has been successfully utilized in the treatment of CIVDH [11–15]. Currently, PECD is mainly divided into the anterior intervertebral approach and the posterior interlaminar approach. We first performed a retrospective comparative study targeting these two approaches.

In this study, the clinical efficacy between these two was not significantly different; however, in the subsequent follow-up, the loss of IVS might be higher after ATd-PECD [15]. It was possible that the removal of the herniated disc through the disc space could also inevitably affect the surrounding nucleus pulposus tissues, thus further aggravating the degeneration of the intervertebral disc. Compared to the transdiscal surgery, the anterior transcorporeal operation is superior to avoid the unrepairable damage to the residual disc. Additionally, the bone defect within the drilling tunnel could be repaired by osteogenesis. This explained why less decrease of the mean height of IVS was detected in the ATc-PECD group. However, the collapse of the superior endplate was observed in two cases. Additionally, the damage to vertebral bone tissue was also controlled. In the other 33 patients, while there was more decrease in the height of the vertebral body, the collapse of the superior endplate did not occur when we controlled the center diameter of drilling hole to approximately 6 mm. This diameter was chosen as our previous finite element analysis, which proved that there was no significant difference in stress distribution when the tunnel diameter was limited to 6 mm [18]. Additionally, the bone wax mixed with methylene blue can mark the ideal puncture trajectory, and thus, the bony tunnel could be accurately and safely created with controlled injury to the vertebral body. Anatomically, the disc herniation was usually located at the same level of intervertebral disc. The transdiscal decompression is more convenient than that in the transcorporeal approach. Thus, the question is that whether there was difference in the clinical outcome between the two approaches. In this study, we found that although the longer time was consumed in the ATc-PECD group to create the bony tunnel, the clinical outcome was favorable in both two groups without significant difference. Because dural sac reexpansion was deemed to the indicator to halt the PECD, the neural decompression was equally satisfactory in these two groups.

Because the diameter of endoscopy was 3.7 mm, it was difficult to place the working cannula into the disc space when the vertical height of IVS was <4 mm. For the disc prolapse, it is really intractable to perform the discectomy under endoscopy via the transdiscal approach. Thus, although both approaches can be utilized to treat paracentral and central disc protrusions, the indications of ATc-PECD can be broader. However, we included these patients with a ventral IVS height of ≥4 mm in this study, which could accommodate the working channel. This facilitates comparing the clinical efficacy between the two approaches. We only included the single-level central or paramedian disc herniation and excluded the patients with foraminal stenosis or lateral disc herniation. We believe that the selected type of disc herniation is the best indication for anterior decompression surgery. For the patients with definite instability of cervical spine, the fusion surgery is more suitable rather than the endoscopic decompression operation. This is why we excluded the patients from our study. For the severely obese patients with a short neck, it is really hard to push the visceral sheath to the opposite side; and the risk of intraoperative vascular and esophageal injury is relatively high. Additionally, when the herniation at the C6-C7 level is downward migrated, the tunnel at the C7 vertebral body cannot be easily located under fluoroscopy. The disc herniation accompanying with severe calcification is controversial to address under endoscope, and the risk of spinal cord injury is higher than that without calcification. Thus, we excluded the aforementioned patients from our study.

The clinical symptoms of both groups were significantly improved, especially the arm pain. This is directly related to thorough neural decompression. There was also a slight improvement in neck pain, which may be related to the recovery of neck mobility after surgical decompression. No severe puncture complications such as major vessel or esophagus injury were observed in two groups. We believed it was related with the “2-finger technique” and medication in the puncture process. In the “2-finger technique,” the lateral carotid artery and medial tracheoesophagus were pushed toward the opposite direction and the tissue space between them was enlarged with the assistance of index and middle fingers of the left hand. A stomach tube with an internal metal wire was used to indirectly display the esophagus, allowing anterioposterior fluoroscopy to determine whether the esophagus was pushed toward the opposite side. The postoperative endplate collapse is the main complication after ATc-PECD. Our another study proved that the bone wax mixed with methylene blue can not only aid hemostasis under endoscope but also facilitate the process of drilling tunnels with a satisfactory trajectory [19]. This explained why only the first two patients experience postoperative endplate collapse. In the ATd-PECD group, the significant decrease in IVS was observed as the endplate collapse was not occurred after transdiscal surgery. Because it was also related with the natural degeneration of intervertebral disc, we did not consider the collapse of intervertebral disc space as the complication. This may have an impact on the analysis of postoperative complications. It was notable that the all two patients experienced the recurrence belonged to the ATd-PECD group. Given that the patients of the ATd-PECD group had a greater postoperative collapse of IVS and a higher recurrence rate, the anterior transdiscal surgery can lead to the iatrogenic injury of intervertebral disc and aggravate the degeneration. Although the loss of height of IVS was significantly higher with the transdiscal approach than with the transcorporeal approach, there was no observed obvious instability.

The main limitations of our study include the use of a single surgeon and institution, small case sample, the lack of randomization, and the specific patient selection criteria. Whether the collapse of IVS or vertebral body can be affected by the bone quality of the vertebral body is unknown. The supraselection of patients can lead to selection bias.

## 5. Conclusions

In the 2-year follow-up, there is no significant difference in the clinical outcomes between the 2 approaches. While the longer time was consumed in the ATc-PECD group, the lower rate of disc collapse and recurrence is notable. Additionally, when the center diameter of the tunnel was limited to 6 mm, the bony defect can be healed without the occurrence of the collapse of the superior endplate, and ATc-PECD may be preferable in the endoscopic treatment of CIVDH.

## Figures and Tables

**Table 1:** Summary of demographics, clinical data, and treatment level.

Baseline characteristics	ATd-PECD (*N* = 42)	ATc-PECD (*N* = 35)
Female gender (%)	16 (38)	12 (34)
Mean age, years (range)	41.3 (28–57)	50.3 (28–72)
Mean duration of symptoms, weeks (range)	15 (6–46)	37 (1–61)
Indications for surgery		
Radiculopathy	33	24
Myelopathy	9	11
Treatment level		
C3-C4	3	4
C4-C5	11	12
C5-C6	23	16
C6-C7	5	3

ATd-PECD: anterior transdiscal percutaneous endoscopic cervical discectomy; ATc-PECD: anterior transcorporeal percutaneous endoscopic cervical discectomy.

**Table 2:** Outcomes of clinical and radiographic.

	ATd-PECD			ATc-PECD			*P* value
	Pre	Post	*P* value	Pre	Post	*P* value	
Clinical outcomes							
Operative time	63.5 ± 10.5 mins			87.5 ± 16.8 mins			*P* ^*∗*^ < 0.05
VAS score for arm pain	6.1 ± 0.5	1.1 ± 0.2	*P* ^*∗*^ < 0.001	6.3 ± 0.6	1.1 ± 0.3	*P* ^*∗*^ < 0.001	*P* ^*∗*^=0.783
VAS score for neck pain	3.7 ± 0.1	2.6 ± 0.1	*P* ^*∗*^ < 0.001	3.7 ± 0.1	2.6 ± 0.1	*P* ^*∗*^ < 0.001	*P* ^*∗*^=0.755

Radiographic outcomes							
Height of IVS	6.0 ± 1.5 mm	5.1 ± 1.1 mm	*P* ^*∗*^ < 0.05	6.8 ± 0.4 mm	6.3 ± 0.5 mm	*P* ^*∗*^ < 0.05	*P* ^*∗*^ < 0.05
Height of the surgical vertebral body	15.1 ± 0.4 mm	15.0 ± 0.2 mm	*P* ^*∗*^ > 0.05	15.8 ± 0.5 mm	15.1 ± 0.4 mm	*P* ^*∗*^ < 0.05	*P* ^*∗*^ > 0.05

Pre: preoperative; post: postoperative. ^*∗*^*t*-test.

## Data Availability

The research related data used to support the findings of this study are restricted by the Ethics Committee of the Second Affiliated Hospital of Chongqing Medical University, in order to protect patient privacy. Data are available from Lei Chu for researchers who meet the criteria for access to confidential data.
